# Efficacy of Compound Kushen Injection in Relieving Cancer-Related Pain: A Systematic Review and Meta-Analysis

**DOI:** 10.1155/2015/840742

**Published:** 2015-10-04

**Authors:** Yu-ming Guo, Yi-xue Huang, Hong-hui Shen, Xiu-xiu Sang, Xiao Ma, Yan-ling Zhao, Xiao-he Xiao

**Affiliations:** ^1^China Military Institute of Chinese Materia Medica, 302 Military Hospital, Beijing 100039, China; ^2^Integrative Medicine Center, 302 Military Hospital, Beijing 100039, China

## Abstract

Despite widespread popular use of complementary and alternative medicine (CAM) therapies, a rigorous evidence based on the efficacy of compound kushen injection (CKI) for cancer-related pain is lacking. In this study, we evaluated the efficacy and safety of compound kushen injection and provided information for current or future research and clinical application. Sixteen trials were identified with a total of 1564 patients. The total pain relief rate of CKI plus chemotherapy is better than chemotherapy except for colorectal cancer. The treatment groups achieved a reduction in the incidences of leukopenia and gastrointestinal, hepatic, and renal functional lesion. However, there is paucity of multi-institutional RCTs evaluating compound kushen injection for cancer pain with adequate power, duration, and sham control. The quantity and quality of RCTs are lower so that we still have to boost the research level through scientific design and normative report.

## 1. Introduction

Pain is the major clinical symptom of cancer patients [1–3]. The management of cancer-related pain is the widespread measure for the patients' quality of life. However, little management of western medicine can be effective. Analgesics are a mainstay of pain management, but they may cause undesired effects such as sedation, nausea, constipation, and renal or liver toxicity. Complementary and alternative medicine therapies are used widely instead among cancer patients to improve cancer-related pain [4, 5]. However, few studies have been published in English written journals that report the effectiveness and safety of many commonly used traditional Chinese medicine (TCM) therapies.

Compound kushen injection (CKI) was approved for the treatment of cancer by the State Food and Drug Administration of China more than 20 years ago. It was also known as Yanshu injection, which contains extracts from two herbs, kushen (Radix sophorae flavescentis) and baituling (Rhizoma smilacis glabrae). The primary components are oxymatrine and matrine [6]. CKI limited cancer pain both directly by blocking TRPV1 signaling and indirectly by reducing tumor growth [7]. Nowadays, it has been used extensively throughout China for pain treatment in combination with conventional analgesics, chemotherapy, or radiotherapy. In this paper, clinical studies were reviewed to evaluate the efficacy and safety of compound kushen injection for cancer-related pain and provide information for current or future research and clinical application.

## 2. Methods

### 2.1. Literature Search

Two independent reviewers, including a librarian, conducted a systematic literature search using databases (MEDLINE, Chinese Biomedical Literature Database, China National Knowledge Infrastructure Database, VIP Database for Chinese Technical Periodicals, Wan-Fang Database, and the Cochrane Library), all from time of inception up to December 2014. A text word search was done in the title and abstract for each concept and combined with the subject heading searches (cancer or neoplasm$ [$ indicates truncation], pain, and kushen injection), and then the search was limited to clinical trials. A similar search was done by another investigator independently using PubMed with the search term “cancer pain” and the limits of “clinical trials” and “kushen injection.” Both reviewers also visually scanned the results to manually remove any citations that were obviously irrelevant and also scanned reference lists of the identified articles to identify any additional articles.

### 2.2. Inclusion and Exclusion Criteria

The inclusion criteria were as follows. (1) Randomized controlled trials (RCTs) of patients with cancer-related pain were selected: the experimental group received compound kushen injection and chemotherapy while control group received chemotherapy only. (2) Outcome assessment included pain-related measurements. (3) No language or blinding restriction was used.

The exclusion criteria were (1) reviews, nonclinical studies, and case observations; (2) no RCTs; (3) controlled interventions with TCM therapies as other Chinese herbs or acupuncture; and (4) inability to find the outcome measurements or duplicated citations.

### 2.3. Types of Outcome Measures

The primary outcome was total pain relief rate. The reduction in pain intensity was measured using a numerical rating scale (NRS), visual analogue scale (VAS), or verbal rating scale. The secondary outcomes were quality of life and adverse events at the end of treatment course.

### 2.4. Data Abstraction

The articles that met the inclusion criteria were reviewed by two independent investigators (Yu-ming Guo, and Yi-xue Huang), and relevant data were extracted. Disagreements were resolved by a third reviewer (Xiu-xiu Sang). The methodological quality of RCTs was assessed independently using seven-item criteria according to the Cochrane Collaboration-Cochrane Tool of Risk of Bias. This scale reports adequate sequence generation, allocation concealment, blinding, incomplete outcome data addressed, selective reporting, other biases, and blinding of outcome assessment.

### 2.5. Data Synthesis and Analysis

Data analyses were performed using the statistical package RevMan 5.0 (Cochrane Collaboration). Dichotomous data were presented as risk ratio (RR) with 95% confidence intervals (CI). Heterogeneity among trials was tested using *I* ^2^ test and considered significant when *I* ^2^ was over 50% or *p* < 0.1. The random effect model was used for the meta-analysis if there was significant heterogeneity while the fixed effect model was used when the heterogeneity was not significant.

## 3. Results

### 3.1. Search Results

**Figure 1:**
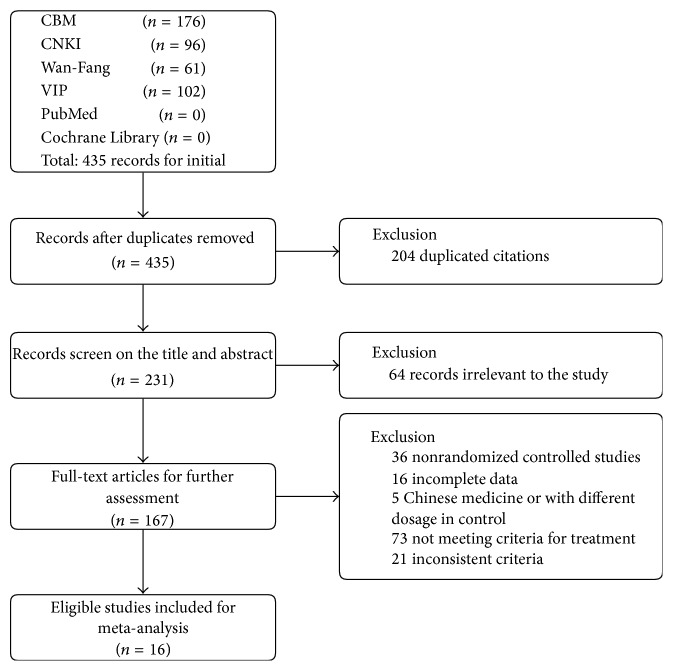
Flow diagram showing the trial selection process for the systematic review and meta-analysis.

A total of 167 references were identified through database searches, of which 151 articles were excluded (36 were not RCTs, 16 did not address the complete data, 5 were associated with other Chinese medicine therapies in experimental group or in control group or with different dosage, 73 did not meet our inclusion criteria for treatment measures, and 21 were with inconsistent criteria), yielding 16 relevant articles for the systematic review and meta-analysis (Figure 1).

### 3.2. Study Characteristics

A total of 1564 study participants were identified from the 16 trials [8–23] (768 were in the control group, 796 were in the treatment group, and the ages ranged from 18 to 85 years). All studies were conducted in China, published between 2006 and 2014. Fifteen trials [8–17, 19–23] were from single institutions and only 1 trial [18] was multicenter RCT. There were different pain scales used among the 16 trials, with the Numerical Rating Scale being the most common. All of the included studies mentioned randomization, but only 4 trials [16, 18–20] reported the method of random sequences generation. No study mentioned allocation concealment. One [19] report recorded the loss to follow-up, and 2 [15, 18] conducted intention-to-treat analysis. Quality assessment of included randomized controlled trials is shown in Table 1.

Six trials [11, 13, 19–21, 23] compared CKI plus FOLFOX to FOLFOX individually. Four trials [12, 15–17] compared CKI plus NP with NP individually. Four trials [8–10, 18] compared CKI plus TACE with TACE individually. Two trials [14, 22] compared CKI plus GC with GC individually. The duration of studies lasted from 10 days to 18 weeks. All studies used the total pain relief rate as primary outcome. The quality of life was reported in 11 studies [8–10, 12, 15–18, 20–23]. Eight trials [8, 11, 12, 14, 16–18, 22] reported adverse effect. Detailed characteristics of included studies are listed in Table 2.

### 3.3. Efficacy Assessment

#### 3.3.1. Total Pain Relief Rate

**Figure 2:**
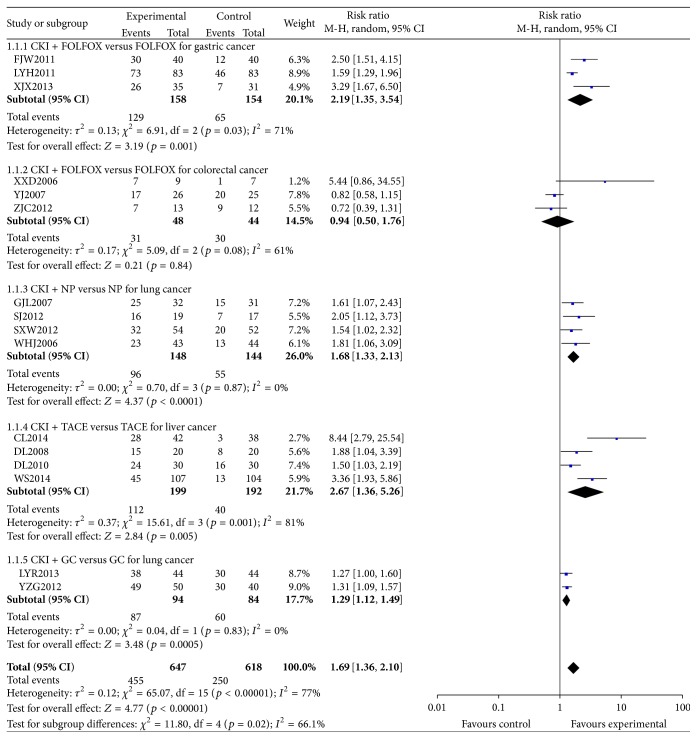
Forest plot of comparison: CKI plus chemotherapy versus chemotherapy alone: total pain relief rate.

All studies adopted the total pain relief rate to assess the improvement for cancer-related pain. The random effect model was used for statistical analysis because heterogeneity was significant (*p* < 0.00001, *I* ^2^ = 77%). The combined effects of 16 independent trial results showed that CKI could relieve pain in patients when compared with chemotherapy (*n* = 1265, RR = 1.69, 95% CI, 1.36 to 2.10, *p* < 0.00001). The subgroup analysis indicated that no better improvements were observed after CKI treatment for colorectal cancer (Figure 2).

#### 3.3.2. Quality of Life

**Figure 3:**
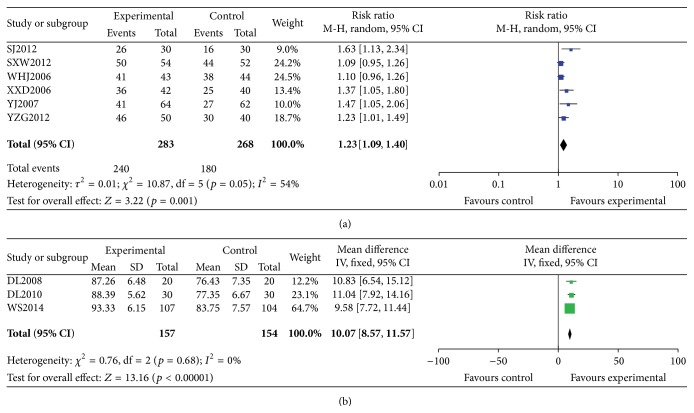
Forest plot of comparison: CKI plus chemotherapy versus chemotherapy alone. (a) KPS increase rate; (b) KPS scores.

Karnofsky performance status (KPS) increase rate data extracted from 6 studies [15–17, 20–22] showed heterogeneity among trials (heterogeneity: *p* = 0.50, *I* ^2^ = 54%). The random effect model was used for statistical analysis. The combined effects of 6 independent trial results showed that CKI had improved the KPS increase rate in patients when compared with chemotherapy alone (*n* = 420, RR = 1.23, 95% CI, 1.09 to 1.40, *p* = 0.001) (Figure 3(a)).

KPS scores extracted from 3 studies [9, 10, 18] showed no heterogeneity among trials (heterogeneity: *p* = 0.68, *I* ^2^  =  0%). The fixed effect model was used for statistical analysis. The combined effects of 3 independent trial results showed that CKI had improved the KPS scores in patients when compared with chemotherapy (*n* = 311, RR = 10.07, 95% CI, 8.57 to 11.57, *p* < 0.00001) (Figure 3(b)).

#### 3.3.3. Adverse Events

**Figure 4:**
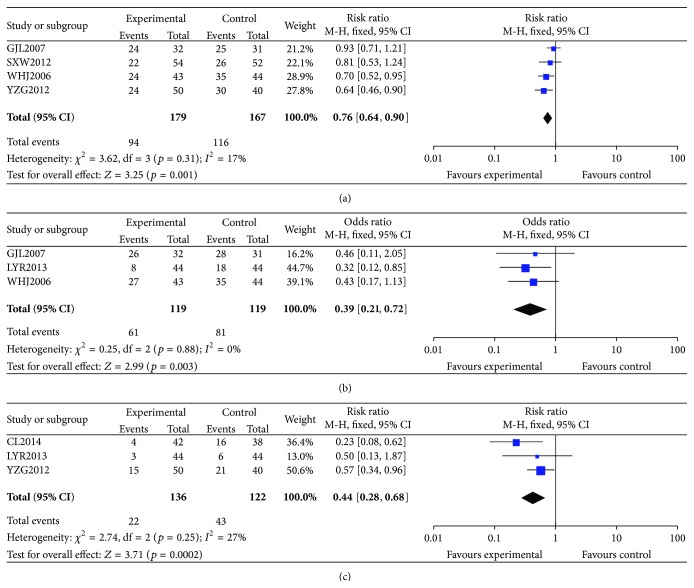
Forest plot of comparison: CKI plus chemotherapy versus chemotherapy alone. (a) Incidences of leukopenia; (b) incidences of gastrointestinal adverse reactions; (c) incidences of hepatic and renal functional lesion.

Specific adverse effects included leukopenia, gastrointestinal adverse reactions, alopecia, hepatic and renal functional lesion, and bone marrow depressions. Of the 16 trials, 8 trials reported adverse effects. The treatment groups achieved a statistically significant reduction in the incidences of leukopenia (*n* = 346, RR = 0.76, 95% CI, 0.64 to 0.90, *p* = 0.001) (Figure 4(a)), gastrointestinal adverse reactions (*n* = 238, RR = 0.39, 95% CI, 0.21 to 0.72, *p* = 0.003) (Figure 4(b)), and hepatic and renal functional lesion (*n* = 258, RR = 0.44, 95% CI, 0.28 to 0.68, *p* = 0.0002) (Figure 4(c)). No severe adverse events were found in the treatment groups.

## 4. Discussion

Cancer-related pain is the perception of the effect of therapy, disease status, quality of services, and even survival [24]. Intractable cancer pain resistant to World Health Organization (WHO) analgesic ladder afflicts 10~15% of cancer pain patients [25]. Patients used to accept analgesics as per WHO analgesic ladder but were found to be recalcitrant or developed intolerable side effects limiting their use or dose. CAM is used as an adjunct therapy with standard pain management techniques as it is noninvasive and generally considered to be relatively free of toxicity [26]. CKI is a mixture of natural compounds extracted from kushen and baituling. It exhibits a variety of pharmacological activities, including anti-inflammatory, antiallergic, antiviral, antifibrotic, and especially anticancer activities [27]. Now with the more and more published RCTs of CKI on cancer-related pain, it is necessary to use the methods of systematic review and meta-analysis to summarize the available evidence and give suggestions to future research and practice. The existing systematic review and meta-analysis assessed the efficacy of CKI only for bone cancer pain or hepatocellular carcinoma [28, 29]. In our study, we used subgroup meta-analysis to evaluate the improvement for cancer-related pain of different organs and assessed the adverse events.

One hundred and sixty-seven studies reviewed were CKI plus chemotherapy in treating cancer-related pain. Sixteen trials were identified as eligible studies for systematic review and meta-analysis. The main findings of the present study were that CKI could improve the total pain relief rate and quality of life of patients with cancer-related pain. Despite the apparent positive findings reported, there is insufficient evidence to support routine use of CKI for cancer-related pain due to the poor methodological quality and the small number of trials of the included studies. Interestingly, the meta-analysis of total pain relief rate about CKI plus chemotherapy for colorectal cancer found no consistent effects with other types of cancer (RR = 0.94, 95% CI, 0.50 to 1.76, *p* = 0.84). There is a clear need for further qualitative and quantitative research to identify the efficacy of CKI for colorectal cancer.

It was of special interest that CKI with chemotherapy could reduce the adverse events. No patients dropped out of their test trial due to the adverse events of CKI, which indicated that CKI is safe for clinical use. As we all know, leukopenia, gastrointestinal adverse reactions, alopecia, and bone marrow depressions are the common side effects of chemotherapy. CKI plus chemotherapy achieved a statistically significant reduction in the incidences of leukopenia, gastrointestinal adverse reactions, and hepatic and renal functional lesion (Figure 4). It indicated directly that CKI could reduce the toxicity of chemotherapy drugs. That was in conformity with the superiority of TCM in toxicity reduction and efficacy enhancement. None of the 16 studies reported whether there were any adverse events relevant to CKI. However, the evidence is limited to make a conclusion on the issue of safety because only 50% of studies mentioned the adverse effects.

*Limitation*. The primary outcome in this review was total pain relief rate. However, the measure through subjective qualitative scores such as “markedly effective,” “effective,” and “ineffective” is not internationally recognized. In addition, the reduction in pain intensity was measured using different scales such as NRS, VAS, and other measures. So it is uncertain in assessing the outcome. No study of the included trials reported whether any adverse events relevant to CKI were apparent in patients. Thus, all adverse events must be reported by the researchers participating in a clinical trial of CKI in the future.

This systematic review and meta-analysis provides moderate evidence for the effectiveness and safety of CKI as adjuvant therapy for cancer-related pain, and a clinical recommendation cannot be warranted because of the generally low methodological quality of the included studies (Table 1). CKI may have beneficial effects in the improvement of total pain relief rate and quality of life and reduction of side effects. However, current evidence is insufficient to support the efficacy of CKI for cancer-related pain because the included studies were of generally poor quality and had small sample sizes. Future research should focus on methodologically strong RCTs to determine the potential efficacy of CKI. The CONSORT statement [30] should be used as a guideline when designing and reporting RCTs for TCM in the future.

## 5. Conclusions

CKI appears to be able to improve total pain relief and quality of life and seems to have beneficial effects on reduction of side effects in patients compared with chemotherapy alone.

## Figures and Tables

**Table 1:** Quality assessment of included randomized controlled trials.

Included trials	Random sequence generation	Allocation concealment	Blinding	Blinding of outcome assessment	Incomplete outcome data	Selective reporting	Other biases
CL2014	Unclear	Unclear	Unclear	Unclear	Yes	No	Unclear
DL2008	Unclear	Unclear	Unclear	Unclear	No	No	Yes
DL2010	Unclear	Unclear	Unclear	Unclear	No	No	Yes
FJW2011	Unclear	Unclear	Unclear	Unclear	No	No	Unclear
GJL2007	Unclear	Unclear	Unclear	Unclear	No	No	Yes
LYH2011	Unclear	Unclear	Unclear	Unclear	No	No	Unclear
LYR2013	Unclear	Unclear	Unclear	Unclear	No	No	Unclear
SJ2012	Unclear	Unclear	Unclear	Unclear	No	No	Yes
SXW2012	Yes	Unclear	Unclear	Unclear	No	No	Unclear
WHJ2006	Unclear	Unclear	Unclear	Unclear	No	No	Unclear
WS2014	Yes	Unclear	No	Unclear	No	No	Unclear
XJX2013	Yes	Unclear	Unclear	Unclear	Yes	No	Unclear
XXD2006	Yes	Unclear	Unclear	Unclear	No	No	Yes
YJ2007	Unclear	Unclear	Unclear	Unclear	No	No	Unclear
YZG2012	Unclear	Unclear	Unclear	Unclear	No	No	Yes
ZJC2012	Unclear	Unclear	Unclear	Unclear	No	No	Unclear

**Table 2:** Summary of included studies.

Included trials	Subject	Age	Type of cancer pain	Intervention		Outcomes
	(experimental/control)	(years; experimental/control)		Experimental	Control	
CL2014	42/38	Unclear	Liver cancer	CKI + TACE	TACE	Total pain relief rate

DL2008	20/20	51 (28–65)/53 (26–66)	Liver cancer	CKI + TACE	TACE	Total pain relief rate; quality of life; adverse events

DL2010	30/30	51 (37–66)/50 (36–66)	Liver cancer	CKI + TACE	TACE	Total pain relief rate; quality of life

FLW2011	40/40	66/65	Gastric cancer	CKI + FOLFOX	FOLFOX	Total pain relief rate; adverse events

GJL2007	32/31	60 (43,71)	Lung cancer	CKI + NP	NP	Total pain relief rate; quality of life; adverse events

LYH2011	83/83	71.4 (60–83)/72.7 (61–85)	Gastric cancer	CKI + FOLFOX	FOLFOX	Total pain relief rate

LYR2013	44/44	52.8 ± 14.5	Lung cancer	CKI + GC	GC	Total pain relief rate; adverse events

SJ2012	18/14	62.65 (33,81)	Lung cancer	CKI + NP	NP	Total pain relief rate; quality of life

SXW2012	54/52	52.5 (32–73)/53.1 (31–72)	Lung cancer	CKI + NP	NP	Total pain relief rate; quality of life; adverse events

WHJ2006	44/43	54 (33,76)	Lung cancer	CKI + NP	NP	Total pain relief rate; quality of life; adverse events

WS2014	107/104	55.6/54.5	Liver cancer	CKI + TACE	TACE	Total pain relief rate; quality of life; adverse events

XJX2013	60/60	18–75	Gastric cancer	CKI + FOLFOX	FOLFOX	Total pain relief rate

XXD2006	45/44	54 (32,71)	Colorectal cancer	CKI + FOLFOX	FOLFOX	Total pain relief rate; quality of life

YJ2007	64/62	56 (30–78)/60 (33–75)	Colorectal cancer	CKI + FOLFOX	FOLFOX	Total pain relief rate; quality of life

YZG2012	50/40	56 (40,78)/58 (43,76)	Lung cancer	CKI + GC	GC	Total pain relief rate; quality of life; adverse events

ZJC2012	33/33	52.3 ± 6.7	Colorectal cancer	CKI + FOLFOX	FOLFOX	Total pain relief rate; quality of life

CKI: compound kushen injection; TACE: Transhepatic Arterial Chemotherapy and Embolization; FOLFOX: oxaliplatin + calcium folinate + fluorouracil; NP: Navelbine + cisplatin; GC: gemcitabine + carboplatin.
